# Comparative Study on the Influence of Inorganic Pigments on the Thermomechanical and Combustion Performance of Different Elastomer Composites

**DOI:** 10.3390/molecules31162762

**Published:** 2026-08-08

**Authors:** Katarzyna Kirchof, Anna Marzec, Bolesław Szadkowski

**Affiliations:** Institute of Polymer and Dye Technology, Faculty of Chemistry, Lodz University of Technology, Stefanowskiego 16, 90-537 Lodz, Poland; 242192@edu.p.lodz.pl

**Keywords:** elastomer composite, inorganic pigments, earth pigment, thermo-oxidative aging, combustion

## Abstract

Inorganic pigments are widely used in elastomer technology primarily as coloring agents; however, the broader effects of these additives on the structure–property relationships of rubber composites remain insufficiently understood, particularly across different elastomer matrices. This study addresses this gap by systematically evaluating and comparing the effects of zirconium cerulean blue (Pigment Blue 71, PB71) and earth pigment iron ocher (IO) in natural rubber (NR), acrylonitrile–butadiene rubber (NBR), and styrene–butadiene rubber (SBR) composites. The influence of pigment loading (2–10 phr, parts per hundred parts of rubber) on rheometric behavior, crosslink density, mechanical performance, thermo-oxidative aging resistance, and combustion behavior was investigated to determine whether inorganic pigments provide benefits beyond coloration. The incorporation of pigments did not adversely affect vulcanization behavior, with the crosslink density of NR increasing from approximately 1.5 × 10^−5^ mol cm^−3^ for the unfilled vulcanizate to 1.5–2.3 × 10^−5^ mol cm^−3^ for pigment-containing composites, while SBR exhibited an increase from approximately 1.0 × 10^−5^ to 1.5–2.0 × 10^−5^ mol cm^−3^. Selected formulations also exhibited improved tensile strength and enhanced resistance to thermo-oxidative aging. Pyrolysis combustion flow calorimetry (PFCF) demonstrated that the incorporation of 10 phr IO reduced the peak heat release rate (pHRR) by approximately 11% (NR), 10% (NBR), and 3% (SBR), whereas 10 phr PB71 decreased this parameter by approximately 13%, 8%, and 38%, respectively. These findings demonstrate that inorganic pigments can serve as multifunctional colorants, simultaneously improving mechanical performance, thermo-oxidative stability, and fire resistance while maintaining effective coloration.

## 1. Introduction

Elastomeric materials play an indispensable role in modern engineering owing to the unique combination of elasticity, resilience, and chemical resistance offered by these materials. Consequently, such materials are extensively employed in demanding applications, including automotive, construction, electrical engineering, and healthcare. Despite these advantages, the hydrocarbon nature of most elastomers results in inherently high flammability and susceptibility to thermo-oxidative degradation, which remain major challenges limiting long-term performance and safety. Improving the fire resistance of elastomers plays an important role in fire safety, while also extending the durability of elastomer products and promoting environmental protection by reducing the risk of environmental pollution. Numerous studies have examined flame-retardant additives, with fire resistance most commonly achieved using phosphorus-derived compounds [1], temperature-responsive fillers [2], and halogen-based additives [3]. Over the past few decades, halogen-containing fire-retardant systems have found widespread application. However, recent studies have demonstrated that the thermal degradation and burning of these materials cause hazardous fumes and smoke emissions [4,5]. These concerns have led to greater awareness of flame-retardant applications and have stimulated research into more eco-friendly and bio-based solutions. In this context, increasing attention has been given to mineral additives that may provide both functional and protective effects in polymer materials [6,7,8].

A variety of industrial and commercial fields rely on the use of dyes and pigments, such as the paint, construction, paper, ceramic, textile, and plastics industries [9,10]. Pigments are substances responsible for coloration that are introduced into polymer composites by dispersion, where they remain a separate phase within the colored material. Moreover, pigment incorporation can enhance photostability and flame-retardant properties [11,12,13]. Recent studies on pigment technologies are focused on reducing the energy consumption associated with cooling by controlling the absorption and reflection of infrared radiation. The application of pigments can effectively reflect heat from solar radiation [14,15,16,17,18], as well as near-infrared radiation (NIR), which is a significant part of the solar radiation [19]. The use of NIR-reflective additives enables a reduction in surface heating, a key factor in automotive applications that extensively employ elastomeric materials [20,21,22]. Pigments can be classified according to origin, distinguishing between natural and synthetic types, or based on chemical nature, allowing classification into organic and inorganic groups. Pigments exhibit minimal migration and are largely insoluble in the majority of solvents, which is advantageous for practical applications. Organic pigments are generally characterized by high color intensity and brightness but often exhibit limited thermal and photochemical stability [23]. Some organic pigments, such as phthalocyanines, have been shown to improve the thermal stability of elastomers and significantly reduce their flammability. These pigments may even impart self-extinguishing behavior under ambient conditions [24,25]. Nevertheless, the synthesis of these pigments is generally complex and time-consuming. Moreover, it often relies on hazardous solvents, which raises both environmental and economic concerns [26]. These limitations have stimulated growing interest in inorganic pigments as more sustainable multifunctional alternatives.

Inorganic pigments can be obtained by straightforward chemical reactions (especially oxidation) or occur naturally in the form of earths. Earth pigments are a group of natural, inorganic mineral raw materials that have been used as colorants for centuries. These materials are characterized by wide availability, good covering properties, and high color fastness and stability, making them an ideal choice for a variety of applications [27]. Iron oxides constitute a broad class of mineral pigments that includes earth pigments, ochres, and iron ores. This group is commonly classified according to color, such as yellow and red ochres or umbers. Iron oxide-based pigments can occur both as independent oxide phases and as chromogenic components present in the clay structure [28]. Recently, these systems have gained considerable attention due to high color intensity, low cost, ease of application, and a variety of useful features [26]. Beaugendre et al. [29] reported that a 200 μm iron oxide-containing coating achieved a UL-94 V0 classification and a limited oxygen index (LOI) of 32 vol%, with no deterioration in fire performance following ultraviolet radiation (UV) and hygrothermal weathering. In a different approach, Camilbel et al. [30] reported that the use of iron ores as an additive in polyacrylate-based coatings led to pronounced improvements in fire performance as well as UV shielding and antibacterial properties. It was reported that increasing the iron ore concentration in the coatings to 5 wt% led to a marked improvement in UV protection, with hematite-, goethite-, and goethite/limonite/hematite-based coatings reaching impressive ultraviolet protection factor (UPF) values of 35, 40, and 50, respectively, indicating very good UV-blocking efficiency. Moreover, Szadkowski et al. [31] demonstrated that natural clay minerals can serve as effective carriers for pigment systems in ethylene–norbornene copolymers, leading to reduced flammability and improved thermal stability. Likewise, El-Nashar et al. [32] reported that micronized phosphate pigments acted as reinforcing colorants for NR/SBR rubber blends, increasing the tensile strength to 20.0–23.4 MPa compared with 18.0 MPa for carbon black-filled and 15.1 MPa for silica-filled vulcanizates. These findings indicate that selected inorganic pigments may simultaneously provide coloration and reinforcement, highlighting the potential of such pigments as multifunctional additives in elastomer technology.

Spinel pigments are mixed metal oxides with the general formula AB_2_O_4_, exhibiting high resistance to sunlight, elevated temperatures, and environmental factors [30]. In the spinel structure (A^2+^B^3+^_2_O_4_), divalent and trivalent metal ions occupy different coordination sites, while in inverse spinels (B[AB]O_4_), the A cations are located in octahedral positions, and the B cations occupy both tetrahedral and octahedral sites. These materials are widely used as catalytic agents, semiconducting materials, and magnetic ceramic powders and are most commonly applied in ceramics as coloring agents [33]. It is worth mentioning that the presence of mineral particles affects the properties of the material as a composite system. The incorporation of inorganic pigments into elastomers not only serves a coloring function but also induces structural and performance-related changes in the material, influencing thermal stability, thermo-oxidative aging behavior, and combustion characteristics [26,34]. Al-Sehemi et al. [35] showed that spinel pigments in SBR elastomers provide both functional and aesthetic benefits. At higher loadings (75–100 phr), the composites maintained stable performance and were suitable for use as substrates and insulating layers in flexible antennas, highlighting the potential of these materials for multifunctional applications. The available literature also highlights zirconium cerulean blue (Pigment Blue 71) as a promising inorganic pigment. This zircon-based ceramic pigment belongs to the family of complex inorganic colored pigments (CICPs) and is characterized by excellent thermal, chemical, and photochemical stability, making it an attractive colorant for high-performance polymer materials [36]. Our previous studies [26] focused on the effect of earth pigments on the aging process and combustion properties of ethylene–norbornene copolymers, showing that pigment incorporation improves resistance to external conditions and provides attractive coloration. Further analysis confirmed that natural earth pigments can act as stabilizers, enhancing mechanical durability and resistance to degradation under sunlight. This was reflected in a high aging factor (AF) remaining close to 1 even after 500 h of irradiation for composites containing earth pigments. The examples presented above demonstrate the wide diversity of inorganic pigments and highlight the versatile influence of these additives on the properties of polymer-based composites.

Despite the growing interest in multifunctional mineral pigments, a systematic comparison of earth pigments and spinel pigments in sulfur-cured elastomers has not yet been reported. In particular, there is insufficient understanding of how pigment chemistry and concentration influence curing characteristics, crosslink density evolution, thermo-oxidative stability, mechanical performance, and combustion behavior across different elastomer matrices. Addressing these knowledge gaps is essential for the rational design of colored elastomer composites exhibiting simultaneously high durability, improved fire performance, and attractive appearance. Therefore, the objective of the present work was to perform a comprehensive comparative investigation of iron ocher and zirconium cerulean blue pigments incorporated into NR, NBR, and SBR matrices.

## 2. Results

### 2.1. Curing Behavior

**Figure 1:**
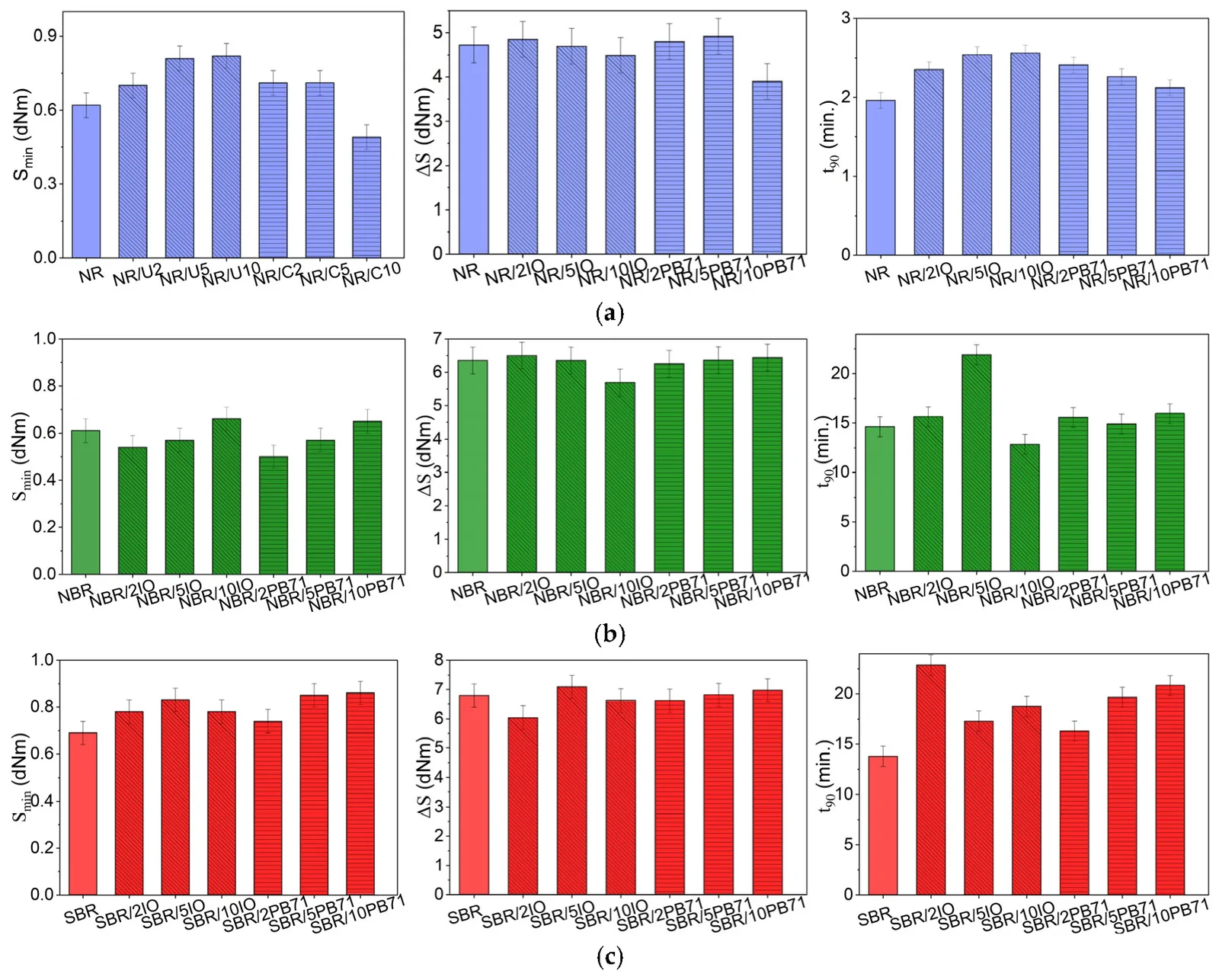
Minimum torque moment, increase in torque moment, and optimum curing time determined in rheometric measurements for NR (**a**), NBR (**b**), and SBR (**c**) composites containing inorganic pigments.

Rheometric measurements provide valuable insight into the processing behavior and curing kinetics of elastomer composites. Figure 1 presents the minimum torque (S_min_), torque increment (ΔS), and optimum curing time (t_90_) for NR-, NBR-, and SBR-based composites containing iron ochre or Pigment Blue 71 at different loadings.

The minimum torque reflects the viscosity and processability of the uncured rubber compounds. The incorporation of inorganic pigments influenced S_min_ to different extents depending on the elastomer matrix, as well as the type and concentration of the pigment. In many cases, an increase in S_min_ relative to the unfilled reference compounds suggested restricted polymer chain mobility due to the presence of rigid filler particles; however, this trend was not observed consistently across all formulations. This effect became more pronounced, often with increasing pigment concentration. For NR and SBR elastomers (Figure 1a,c), the addition of IO led to a more substantial rise in S_min_ than PB71 at equivalent loadings, suggesting a higher tendency of IO particles to form physical networks within these rubber matrices. Iron ochre, primarily composed of iron oxides, possesses a polar surface capable of interacting with non-rubber components of the compound, such as stearic acid and zinc oxide, which may contribute to increased compound stiffness. On the other hand, for the NBR/2IO, NBR/5IO, NBR/2PB71, and NBR/5PB71 composites (Figure 1b), the S_min_ values were generally slightly lower than those of the reference compound, with an increase observed only at the highest pigment loading (NBR/10IO and NBR/10PB71). This trend may result from the relatively weak interactions between the pigment particles and the NBR matrix at low concentrations, while the higher pigment content increased the resistance to deformation due to the greater volume fraction of solid particles.

The torque increment, defined as the difference between the maximum and minimum torque, is commonly associated with crosslink density and the reinforcing efficiency of fillers. Across all elastomer systems, ΔS did not exhibit significant changes in the presence of inorganic pigments, even at higher pigment loadings. In all investigated formulations, ΔS remained within comparable ranges (~4.5 dNm for NR, ~6.0 dNm for NBR, and ~7.0 dNm for SBR), indicating that the applied inorganic pigments have no substantial effect on the crosslinking process of these elastomers. Only in the case of NR containing 10 phr of pigment PB71 was a slight decrease in ΔS observed, reaching values below 4.0 dNm. This reduction may be associated with the influence of the higher pigment loading on vulcanization kinetics, resulting from local filler–rubber interactions and/or less efficient distribution of the curing system within the rubber matrix. The optimum curing time is a key parameter describing vulcanization kinetics. In general, pigment addition influenced t_90_ differently depending on pigment type and elastomer matrix. In the case of NR-based composites (Figure 1a), the addition of inorganic pigments had no significant effect on the curing time. The optimum vulcanization time remained very short, ranging from approximately 2.0 to 2.5 min for all NR formulations, regardless of pigment type or concentration. A slight prolongation of t_90_ was observed for NBR composites upon pigment incorporation (Figure 1b). A particularly pronounced effect was recorded for the NBR composite containing 5 phr iron ochre, where the curing time increased markedly from about 15 min for the unfilled material to over 20 min. The prolonged curing time suggests that iron oxide particles may interfere with the vulcanization process through filler–curative interactions, possibly due to the adsorption of accelerators onto the particle surface. For SBR-based composites (Figure 1c), the incorporation of inorganic pigments also resulted in a moderate extension of curing time. The increase in t_90_ became more evident with rising pigment content, reaching approximately 22 min for the SBR/2IO composite and about 20 min for SBR/10PB71. These results confirm that inorganic pigments exert a stronger retarding effect on vulcanization kinetics in SBR and NBR matrices than in NR.

### 2.2. Mechanical Properties

**Figure 2:**
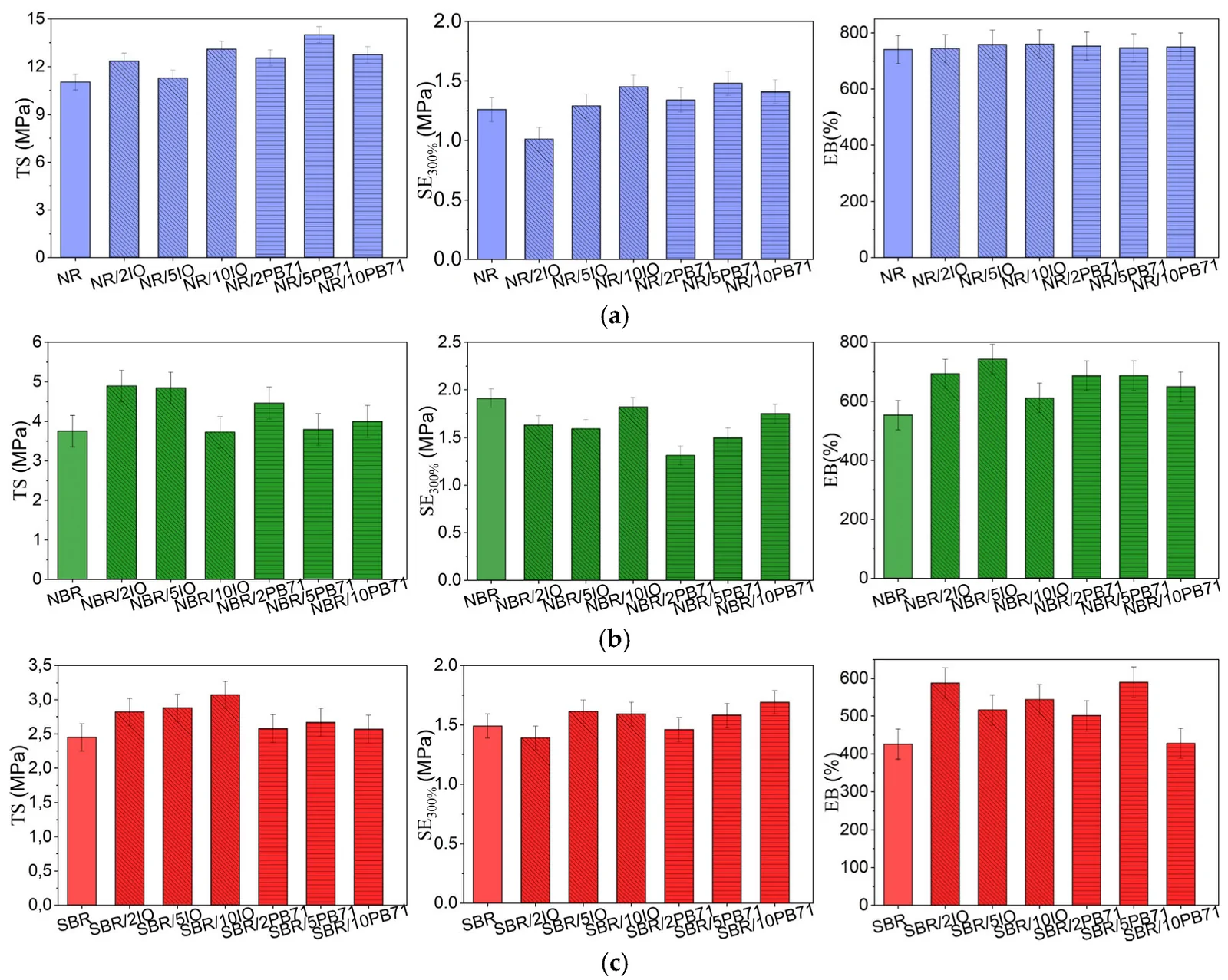
Tensile strength, stress at 300% deformation, and elongation at break determined in tensile tests for NR (**a**), NBR (**b**), and SBR (**c**) composites containing inorganic pigments.

The mechanical performance of the elastomeric composites was evaluated under static tensile loading, and the obtained results for the investigated elastomers are summarized in Figure 2.

As expected, significant differences in tensile strength (TS) were observed among the reference materials. The lowest TS was recorded for styrene–butadiene rubber (SBR, 2.5 MPa), followed by nitrile–butadiene rubber (NBR, 3.8 MPa), while natural rubber exhibited markedly superior strength, reaching 11 MPa. The considerably higher TS of NR compared to SBR and NBR can be attributed primarily to its unique microstructure and strain-induced crystallization ability. Natural rubber consists predominantly of cis-1,4-polyisoprene units, which enable effective chain alignment and crystallization under tensile deformation, thereby significantly enhancing load transfer and resistance to crack propagation. In contrast, SBR and NBR are amorphous elastomers lacking this crystallization mechanism, which limits the ultimate tensile performance of these materials [37]. Overall, the incorporation of the inorganic pigments investigated resulted in relatively small changes in tensile strength (TS), and no consistent trend was observed across all elastomer systems. Nevertheless, the response depended on both the elastomer matrix and the pigment type. In the case of NR-based composites, the addition of 2 phr and 10 phr of IO increased TS to values exceeding 12 MPa. A beneficial effect of IO on tensile strength was also observed in our previous study, where its incorporation into the cyclic olefin copolymer (COC) increased TS from 40.0 MPa to 41.4 MPa [26]. An even greater improvement was obtained for the NR composite containing 5 phr of PB71, which exhibited the highest TS among all NR formulations, indicating the good reinforcing efficiency of this pigment in the natural rubber matrix. For SBR composites, a gradual increase in tensile strength was observed with increasing IO content, indicating a concentration-dependent reinforcing effect of this pigment within the SBR matrix. Tensile tests revealed that the neat SBR sample exhibited a TS of 2.5 MPa, while incorporation of 2, 5, and 10 phr of IO resulted in TS values of 2.8, 2.9, and 3.2 MPa, respectively. For SBR composites filled with PB71, the TS values ranged from 2.6 to 2.8 MPa. In contrast, the NBR system exhibited a non-monotonic response. Although the addition of 2 and 5 phr of IO enhanced TS, a further increase to 10 phr reduced the tensile strength below that of the reference sample (3.7 MPa). This behavior is more likely associated with the higher pigment loading and its influence on the sulfur vulcanization process. The presence of metal-containing pigments may alter curing kinetics, leading to changes in crosslink density that consequently affect the mechanical performance of the vulcanizates. Although pigment dispersion could also contribute to the observed behavior, no direct morphological evidence is available to confirm such an effect. Likewise, the incorporation of PB71 into SBR and NBR composites did not produce significant changes in TS, suggesting that its reinforcing effect in these elastomer matrices was limited under the investigated conditions. Interestingly, the application of the investigated inorganic pigments had no discernible effect on the elasticity of NR, with elongation at break remaining consistently around 700%. In contrast, for both SBR and NBR composites, the presence of pigments contributed to an increase in elongation at break. For instance, the addition of 2 and 5 phr of IO pigment increased the elongation at break (EB) of NBR from approximately 580% to about 690% and 780%, respectively, while PB71 increased the EB to approximately 650–700%. These changes may also be associated with pigment-induced modifications of the vulcanization process and the resulting crosslinked network structure, which can influence the balance between stiffness and chain mobility. However, further studies involving direct characterization of the crosslink network would be required to confirm this mechanism.

### 2.3. Thermo-Oxidative Aging

**Figure 3:**
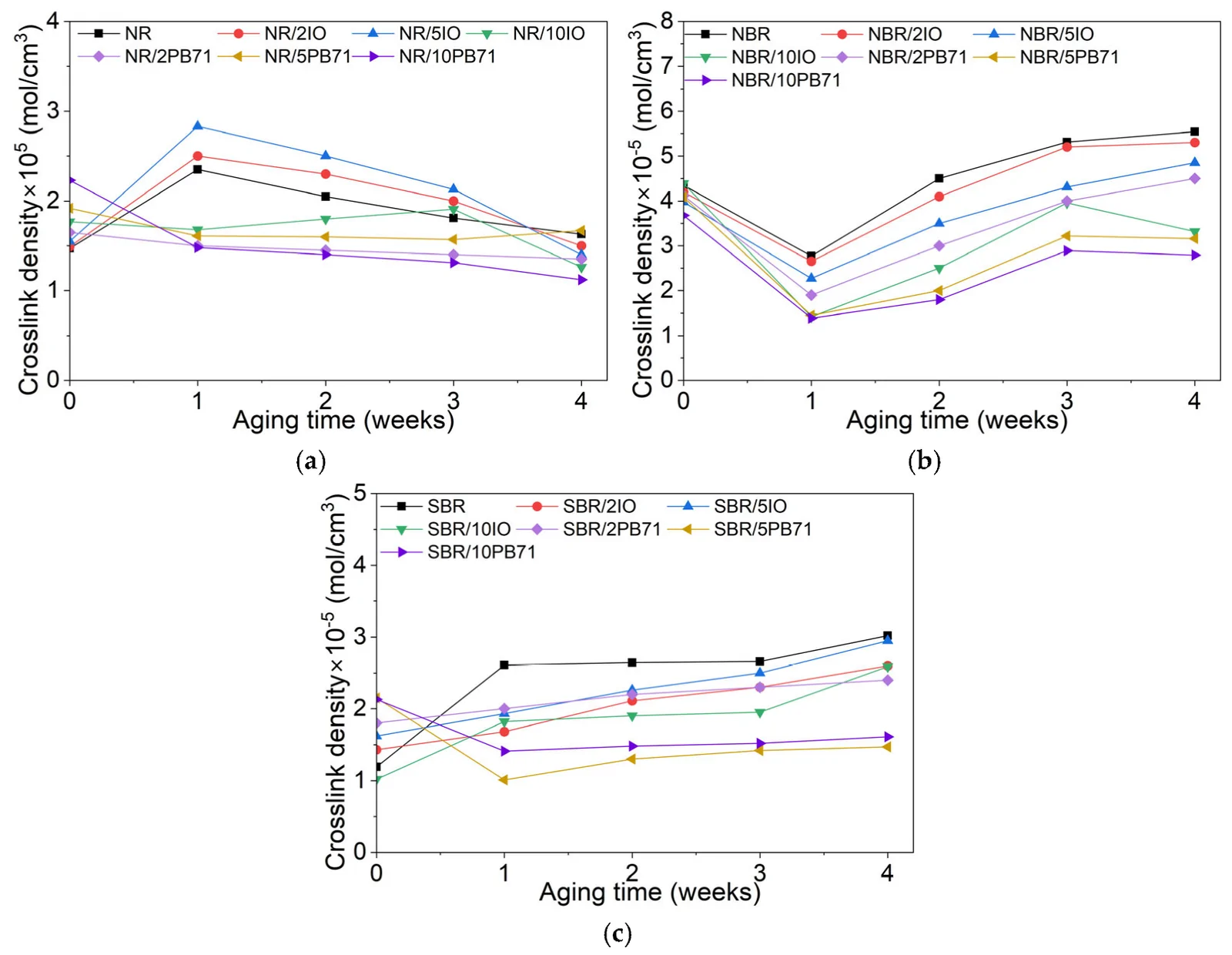
Crosslink density of the vulcanizates after 1, 2, 3, and 4 weeks of thermo-oxidative aging determined for NR (**a**), NBR (**b**), and SBR (**c**) composites containing inorganic pigments.

**Figure 4:**
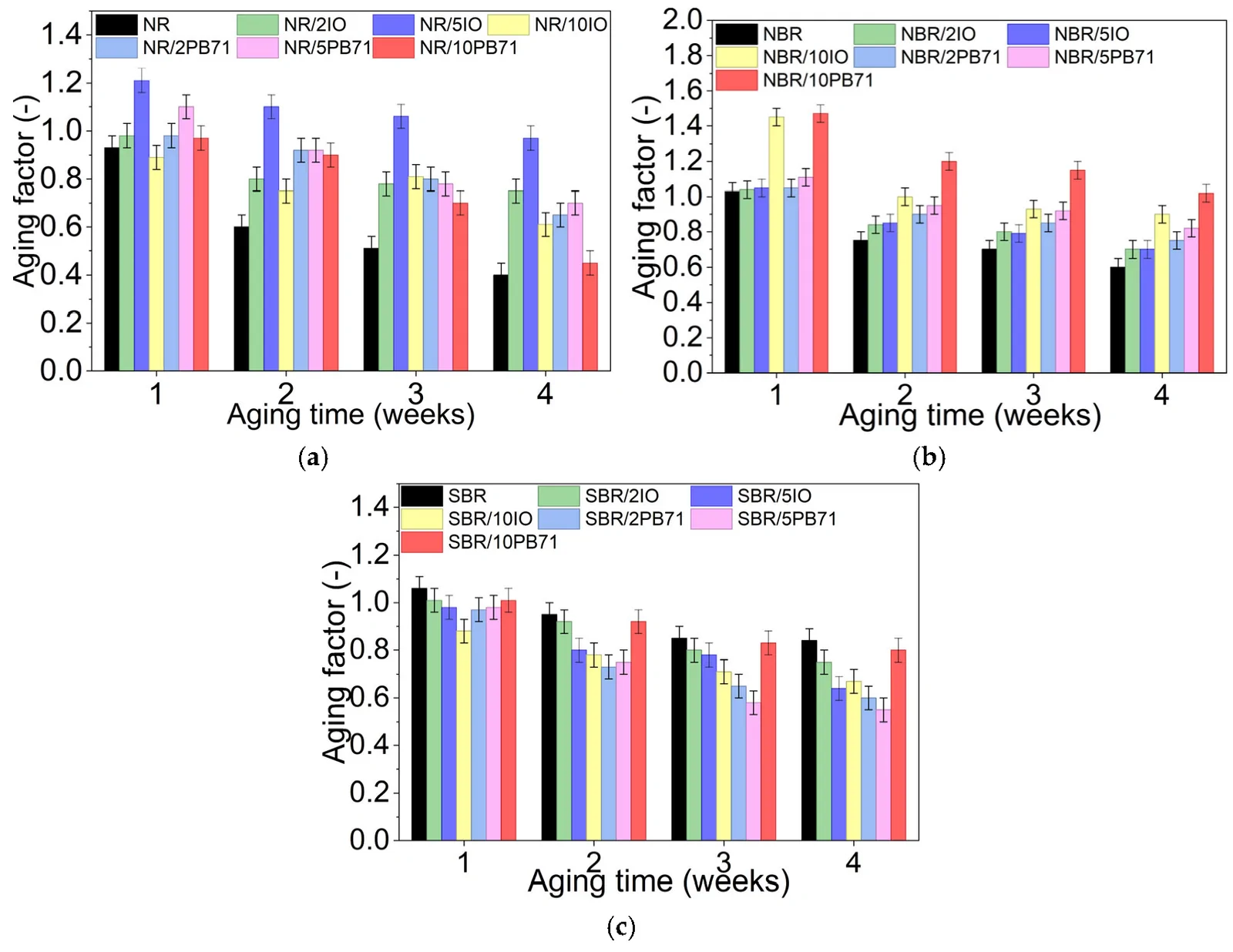
Mechanical aging factor (AF) of the vulcanizates after 1, 2, 3, and 4 weeks of thermo-oxidative aging determined for NR (**a**), NBR (**b**), and SBR (**c**) composites containing inorganic pigments.

The elastomeric composites filled with inorganic pigments were subjected to thermo-oxidative aging at 70 °C for four weeks, during which changes in crosslink density (Figure 3) and the corresponding evolution of mechanical properties expressed by the aging factor (Figure 4) were systematically monitored.

Prior to thermo-oxidative aging (0 weeks), the incorporation of inorganic pigments generally resulted in a slight increase in the initial crosslink density of the NR- and SBR-based composites compared with the corresponding unfilled references (Figure 3a,c). For NR, the initial crosslink density increased from approximately 1.5 × 10^−5^ mol cm^−3^ for the unfilled vulcanizate to approximately 1.5–2.3 × 10^−5^ mol cm^−3^, depending on the pigment type and concentration. Similarly, the initial crosslink density of SBR increased from approximately 1.0 × 10^−5^ to 1.5–2.0 × 10^−5^ mol cm^−3^. In contrast, an opposite tendency was observed for the NBR-based composites (Figure 3b). For both reference NR and those containing 2 and 5 phr of IO pigment, a similar aging-induced trend in crosslink density was observed. After one week of thermo-oxidative exposure, a modest increase in crosslink density occurred, followed by a gradual decrease upon prolonged aging. The initial increase in crosslink density may be attributed to post-curing reactions and additional crosslink formation promoted by elevated temperature and oxidative conditions [38]. However, continued aging leads to the predominance of oxidative chain scission and network degradation, resulting in an overall reduction in crosslink density after 4 weeks of aging for NR composites. In contrast, NBR composites exhibited reversed behavior. After one week of aging, a pronounced decrease in crosslink density was observed, from approximately 4 × 10^−5^ mol cm^−3^ to about 1.5–3 × 10^−5^ mol cm^−3^ (Figure 3b). Further thermo-oxidative exposure led to a gradual increase in crosslink density for all NBR samples, suggesting the dominance of secondary crosslinking reactions at extended aging times, likely associated with the specific polarity and oxidative sensitivity of the nitrile rubber matrix. For the SBR-based composites (Figure 3c), the unfilled reference exhibited a pronounced increase in crosslink density after the first week of thermo-oxidative aging (similar to NR), followed by a plateau, ultimately reaching a value more than twice as high as the initial one. In contrast, the SBR composites containing the IO pigment showed a gradual increase in crosslink density throughout the aging period, indicating the progressive formation of additional crosslinks during thermo-oxidative exposure. Conversely, the incorporation of the PB71 pigment resulted in a sharp initial decrease in crosslink density after the first week of aging, followed by stabilization at nearly constant values for the remainder of the experiment. This behavior suggests that the presence of PB71 initially promoted chain scission or disruption of the crosslinked network, whereas further degradation and crosslinking reactions reached a dynamic equilibrium during prolonged aging.

The evolution of mechanical properties during thermo-oxidative aging, quantified by the aging factor, is presented in Figure 4 and Table 1. The data clearly indicate a progressive aging process for all investigated elastomeric systems, manifested by a gradual reduction in AF with increasing aging time. The most pronounced deterioration was observed for NR-based composites, where AF decreased from approximately 0.9 to 0.4 after four weeks of exposure (Figure 4a). This decreasing trend is in good agreement with previous reports on the thermo-oxidative aging of NR elastomer composites [39,40]. This severe decline can be attributed to the high susceptibility of natural rubber to oxidative degradation, leading to extensive chain scission and a substantial loss of load-bearing capability under prolonged thermal and oxidative conditions. Importantly, the incorporation of inorganic pigments exerted a beneficial effect on the retention of mechanical performance during aging. Among the investigated NR-based formulations, both composites containing iron ocher (NR/2IO and NR/5IO) exhibited favorable resistance to thermo-oxidative aging, as evidenced by the relatively small variation of the aging factor during the entire exposure period (ΔAF = −0.23 and −0.24, respectively). The NR/5IO compound exhibited a relatively stable aging behavior, with AF values of approximately 1.2 after one week, around 1.1 after two and three weeks, and 0.97 after four weeks of aging. Although the AF value slightly decreased below unity after prolonged exposure, the combined tensile strength and elongation at break remained close to those of the unaged material, indicating limited deterioration of the elastomer network. This behavior can be attributed to post-curing phenomena and additional crosslink formation occurring during the initial stages of thermo-oxidative exposure, which may compensate for the onset of oxidative degradation. The comparable stability observed for NR/2IO and NR/5IO suggests that iron ocher, at moderate concentrations, contributes to maintaining a stable crosslinked network and delaying oxidative degradation of the NR matrix. Increasing the IO content to 10 phr slightly reduced this beneficial effect, as reflected by a higher decrease in AF values (ΔAF = −0.28); however, this change remained considerably lower than that observed for the unfilled NR reference (ΔAF = −0.52), indicating that the presence of IO still provided a favorable contribution to thermo-oxidative stability even at the highest investigated concentration.

A more formulation-dependent behavior was observed for the NBR- and SBR-based composites (Figure 4b,c). Among the NBR formulations, the incorporation of 10 phr of PB71 resulted in the highest AF values throughout the entire aging period, decreasing from approximately 1.47 after one week to 1.02 after four weeks. A similarly beneficial effect was observed for the composite containing 10 phr of IO, whose AF values decreased from approximately 1.45 to 0.90 over the same period. These formulations therefore exhibited the highest absolute AF values among the NBR compounds, indicating superior retention of mechanical performance compared with the unfilled reference, whose AF decreased from approximately 1.03 to 0.69. However, despite the favorable absolute performance, both high-loading formulations underwent relatively large reductions in AF (ΔAF ≈ −0.45–0.55) during prolonged aging (Table 1). In contrast, NBR composites containing 2 and 5 phr of IO exhibited smaller changes, with AF decreasing from approximately 1.04–1.05 to 0.70, while the formulation containing 2 phr of PB71 showed a moderate decrease from approximately 1.05 to 0.75. These observations indicate that high initial AF values do not necessarily correspond to the smallest deterioration during prolonged thermo-oxidative exposure.

The behavior of the SBR-based composites differed from that of the NBR systems. The unfilled SBR vulcanizate and the composite containing 2 phr of IO exhibited very similar AF values throughout the aging period, decreasing from approximately 1.05 to 0.84 and from 1.01 to 0.75, respectively, indicating that this pigment concentration had only a limited influence on the degradation process. In contrast, the addition of 10 phr of PB71 provided the highest AF values among the pigment-filled SBR composites at each aging stage, with AF remaining close to 1.01 after one week and 0.80 after four weeks, demonstrating the most pronounced stabilizing effect. Conversely, the formulation containing 5 phr of PB71 exhibited the greatest deterioration, with AF decreasing from approximately 0.98 to 0.55, whereas increasing the IO loading from 2 to 10 phr did not result in a proportional improvement in aging resistance, as the AF after four weeks ranged only from approximately 0.67 to 0.75. These findings demonstrate that the stabilizing efficiency of inorganic pigments is determined by pigment concentration, pigment chemistry, and interactions with the elastomer matrix. Moreover, the present results highlight that assessment of thermo-oxidative stability should account for both the absolute AF values and the temporal evolution of AF, because formulations exhibiting the highest initial AF may still show relatively pronounced degradation during prolonged thermo-oxidative exposure.

### 2.4. Combustion

**Figure 5:**
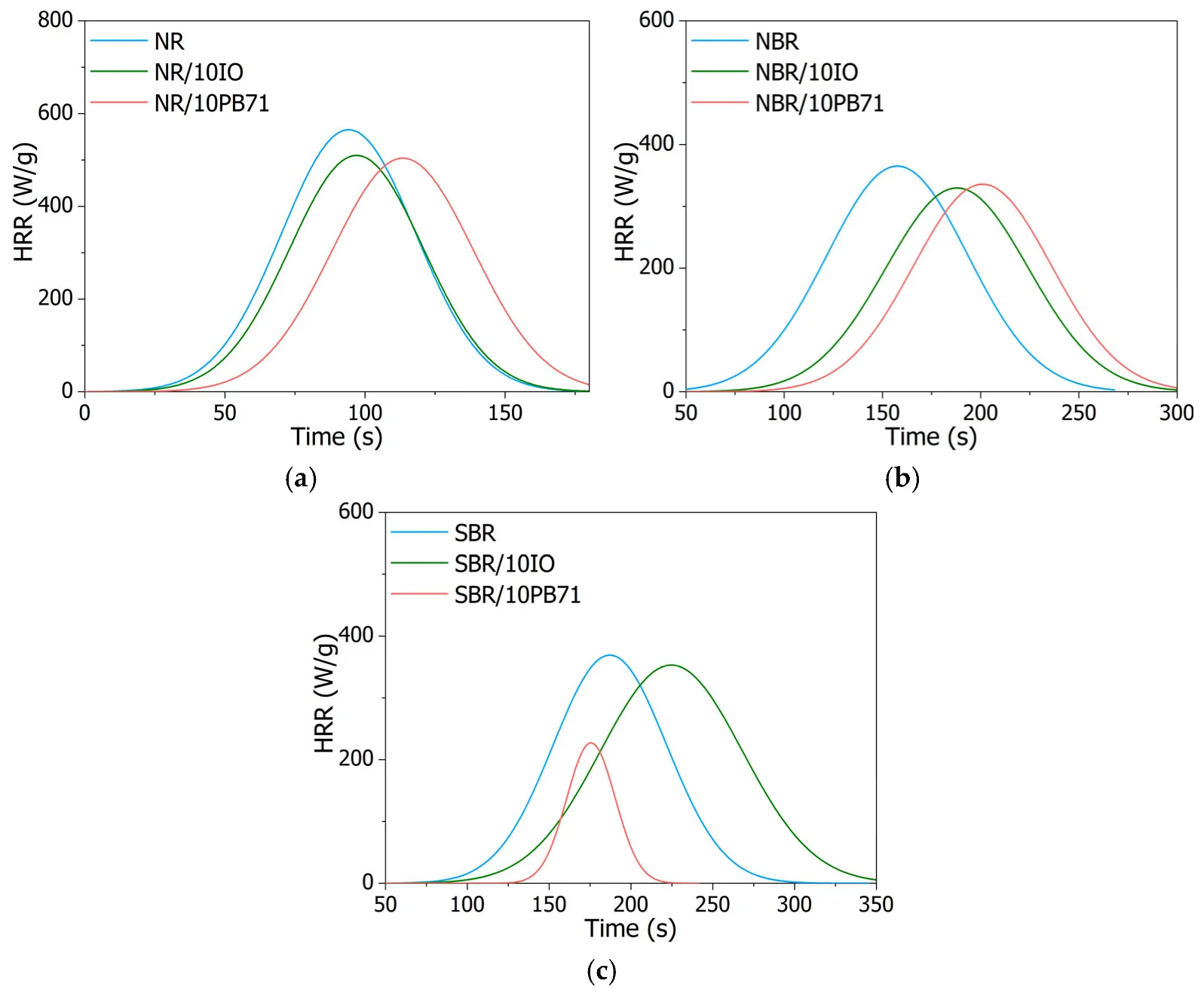
Heat release rate curves determined in PCFC experiments for NR (**a**), NBR (**b**), and SBR (**c**) composites containing inorganic pigments.

The combustion properties of elastomers colored with inorganic pigments were investigated using pyrolysis combustion flow calorimetry. This technique enabled the determination of key flammability parameters such as the heat release rate, total heat release, and heat release capacity, which together provide a comprehensive insight into the combustion behavior and fire susceptibility of the analyzed materials. For this analysis, representative formulations containing the highest pigment loading (10 phr) were selected to evaluate the potential maximum effect of inorganic pigments on flammability-related performance. The results obtained are presented in Figure 5 and Table 2.

Based on the PCFC analysis of the unfilled reference elastomers (neat matrices), natural rubber exhibited the highest flammability, with a maximum HRR value of approximately 578.7 W/g. In comparison, the neat styrene–butadiene rubber and nitrile–butadiene rubber vulcanizates showed considerably lower HRR values of approximately 368.9 and 365.4 W/g, respectively. These values indicate that, among the investigated neat elastomer matrices, NR was inherently the most susceptible to combustion. This trend is consistent with literature data, which indicate that elastomers containing a higher proportion of unsaturated hydrocarbon chains, such as NR, typically exhibit higher heat release rates and greater flammability. In contrast, the presence of polar nitrile groups in NBR contributes to enhanced thermal stability and reduced evolution of flammable volatile products during decomposition, resulting in lower HRR and HRC values [41]. Both inorganic pigments contributed to a reduction in the combustion intensity of the tested elastomers, as confirmed by the analysis of HRR, THR, and HRC parameters. The presence of 10 phr of pigment IO resulted in a reduction in the maximum heat release rate by approximately 11% for NR, 3% for SBR, and 10% for NBR, compared to the unpigmented reference compounds (Figure 5). The influence of iron ochre on the reduction in combustion parameters has previously been observed in cycloolefin copolymer composites. Notably, the incorporation of this pigment resulted in a decrease in the peak heat release rate of approximately 18% compared to the reference material [26]. An even more pronounced effect was observed for pigment PB71. The incorporation of 10 phr PB71 led to a decrease in pHRR of about 13% for NR, 38% for SBR, and 8% for NBR. A similar downward trend was also recorded for THR and HRC values (Table 2), indicating an overall improvement in the fire performance of the analyzed elastomers. These results suggest that both inorganic pigments exhibit a flame-retarding effect, although the magnitude of this improvement depends on the elastomer type and the specific pigment applied.

## 3. Materials and Methods

Three elastomeric matrices were employed in this investigation: natural rubber, styrene–butadiene rubber, and acrylonitrile–butadiene rubber. All rubber grades were supplied by Torimex-Chemicals Ltd. Sp. z o.o. (Konstantynow Lodzki, Poland). The vulcanization system consisted of sulfur (S) as the curing agent, mercaptobenzothiazole (MBT) as the accelerator, stearin as the activator, and zinc oxide as the co-activator; all components were sourced from the same supplier. The elastomeric composites were modified by the incorporation of inorganic pigments used as colorants. Iron ocher (IO, 40301, Kremer Pigmente, Aichstetten, Germany) and zirconium cerulean blue (Pigment Blue 71, PB71, Kremer Pigmente, Aichstetten, Germany) were used in this study. IO is a natural iron oxide-based pigment composed mainly of hydrated iron(III) oxides (FeOOH/Fe_2_O_3_) with minor mineral admixtures depending on its geological origin. PB71 is an inorganic zirconium silicate-based pigment (ZrSiO_4_:V), belonging to vanadium-doped zirconium cerulean blue pigments. Both pigments are characterized by high thermal and chemical stability, low solubility, and good resistance to environmental degradation, which makes them suitable for elastomer processing under elevated temperatures. The pigments were incorporated into the rubber matrices at loadings of 2, 5, and 10 phr.

**Figure 6:**
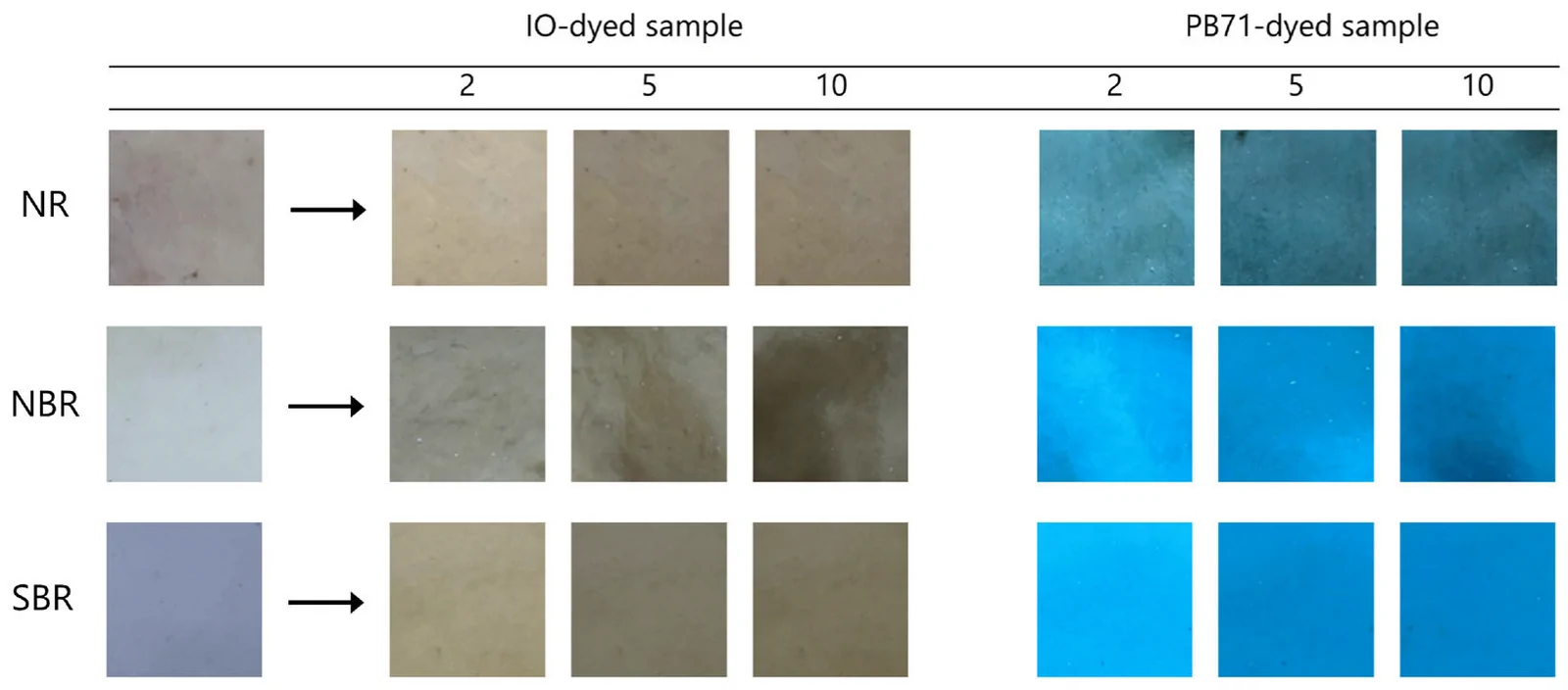
Images of the elastomer composites filled with IO and PB71 inorganic pigments.

Rubber compounds were prepared using a laboratory-scale two-roll mill (David Bridge & Co., Rochdale, UK) fitted with rolls of 200 mm in diameter and 450 mm in length. The rotational speed of the front roll was adjusted to 16 rpm, while a friction ratio of 1:1.2 was applied. During compounding, the nip gap was controlled within the range of 1.5–3 mm. The mixing process was carried out for 10 min at a temperature of approximately 30 °C. Detailed compositions of the investigated compounds and corresponding designations are provided in Table 3, while images of the resulting multicolor elastomer composites are depicted in Figure 6.

The curing behavior of the elastomer composites was analyzed at 160 °C in accordance with ISO 6502 guidelines [42], employing a D-RPA 3000 rotorless rheometer (MonTech, Buchen, Germany). The rheometric analysis enabled determination of key vulcanization parameters, including the minimum torque, maximum torque, and the optimum curing time. The curing process was performed on an electrically heated hydraulic press (Skamet 54436, SKA-MET, Skarzysko-Kamienna, Poland) at 160 °C with 15 MPa of pressure for a curing time consistent with the vulcanization parameters. Mechanical performance under tensile loading was assessed following the ISO 3724 standard [43] using a Zwick Roell 1435 universal testing machine (Ulm, Germany). For each vulcanizate plate, five dumbbell-shaped specimens were tested. The samples were approximately 1 mm thick and 40 mm wide. Tensile measurements were conducted at a constant crosshead speed of 500 mm/min. The crosslink density of the vulcanized materials was determined using the equilibrium swelling technique in accordance with PN-ISO 1817:2021 [44]. This method relies on quantifying mass changes of samples immersed in a solvent, namely toluene. Four specimens with masses ranging from 20 to 50 mg were cut from each vulcanizate. The samples were placed in sealed weighing vessels, fully immersed in the solvent, and allowed to swell for 48 h. Subsequently, they were weighed, dried in a laboratory oven at 50 °C for an additional 48 h, and reweighed after drying. The crosslink density values were calculated using the Flory–Rehner equation (Equation (1)) [45]:

(1) $$\upsilon_{e}=-\frac{\ln\left(1-V_{r}\right)+V_{r}+µ\cdot V_{r}^{2}}{V_{0}\cdot \left(V_{r}^{\left(\frac{1}{3}\right)}-\frac{V_{r}}{2}\right)},$$

where υ*_e_* is the crosslink density of vulcanizates, *V_r_* is the volume fraction of the elastomer in swollen gel, and *V*_0_ is the molar volume of the solvent [mol/cm^3^]. Thermo-oxidative aging resistance of the elastomeric vulcanizates was examined following the procedure specified in ISO 188 [46]. Vulcanized composite plates with a thickness of approximately 1 mm were stored in a controlled-air aging oven (Binder, Tuttlingen, Germany) at 70 °C for periods of 1, 2, 3, and 4 weeks. The influence of aging was evaluated by comparing the crosslink density and tensile strength of aged samples with those of unaged references. In addition, the aging factor was calculated using Equation (2), where TS corresponds to tensile strength and EB represents elongation at break [47]:

(2) $$AF=\frac{{TS}_{aged}\cdot \;{EB}_{aged}}{TS\cdot EB}.$$

The combustion behavior of the elastomer composites was assessed using a PCFC microcalorimeter (Fire Testing Technology Limited, East Grinstead, UK). Approximately 2.5 mg samples of each vulcanizate were prepared in the form of comparable fragments for the measurements. During the analysis, the pyrolyzer and combustor temperatures were maintained at 750 °C and 900 °C, respectively. The three specimens from each vulcanizate were subjected to a linear heating rate throughout the experiment.

## 4. Conclusions

This study demonstrates that iron ocher and zirconium cerulean blue can be successfully incorporated into sulfur-cured NR, NBR, and SBR elastomer composites as multifunctional additives rather than solely as coloring agents. The results reveal that the influence of inorganic pigments is governed by both the elastomer matrix and pigment concentration, highlighting the complex nature of pigment–rubber interactions. The pigments did not adversely affect vulcanization behavior, confirming compatibility with conventional sulfur curing systems. Moreover, the incorporation of pigments increased the crosslink density of NR from approximately 1.5 × 10^−5^ mol cm^−3^ for the unfilled vulcanizate to 1.5–2.3 × 10^−5^ mol cm^−3^, while SBR exhibited an increase from approximately 1.0 × 10^−5^ to 1.5–2.0 × 10^−5^ mol cm^−3^, depending on pigment type and concentration. In contrast, the incorporation of inorganic pigments resulted in a slight reduction in the crosslink density of the NBR composites. Selected formulations simultaneously maintained or slightly improved mechanical performance and exhibited enhanced resistance to thermo-oxidative aging. The PCFC analysis further demonstrated the multifunctional character of the investigated pigments. The incorporation of 10 phr IO reduced the pHRR by approximately 11% for NR, 10% for NBR, and 3% for SBR, whereas 10 phr PB71 resulted in reductions of approximately 13%, 8%, and 38%, respectively. These findings indicate that conventional inorganic pigments can improve the fire performance of elastomer composites without the use of dedicated flame-retardant additives. From a scientific perspective, this work provides new insight into the multifunctional role of different kinds of inorganic pigments in elastomer systems and establishes relationships between pigment chemistry, concentration, elastomer matrix, and the resulting curing behavior, network structure, thermo-oxidative stability, mechanical performance, and flammability. The presented results demonstrate that appropriately selected inorganic pigments can simultaneously fulfill coloring, reinforcing, stabilizing, and flame-retardant functions, providing an effective strategy for designing durable and high-performance colored elastomer composites.

## Figures and Tables

**Table 1:** Aging factor evolution during thermo-oxidative aging together with the overall change between Week 1 and Week 4.

Compound	Week 1	Week 2	Week 3	Week 4	ΔAF (W1→W4)
NR	0.93	0.6	0.51	0.4	−0.53
NR/2IO	0.98	0.8	0.78	0.75	−0.23
NR/5IO	1.21	1.1	1.06	0.97	−0.24
NR/10IO	0.89	0.81	0.75	0.61	−0.28
NR/2PB71	0.98	0.92	0.8	0.65	−0.33
NR/5PB71	1.10	0.92	0.78	0.7	−0.4
NR/10PB71	0.97	0.9	0.7	0.45	−0.52
NBR	1.03	0.83	0.8	0.69	−0.34
NBR/2IO	1.04	0.84	0.8	0.7	−0.34
NBR/5IO	1.05	0.85	0.79	0.7	−0.35
NBR/10IO	1.45	1	0.93	0.9	−0.55
NBR/2PB71	1.05	0.9	0.85	0.75	−0.3
NBR/5PB71	1.11	0.95	0.92	0.82	−0.29
NBR/10PB71	1.47	1.2	1.15	1.02	−0.45
SBR	1.05	0.95	0.85	0.84	−0.21
SBR/2IO	1.01	0.92	0.8	0.75	−0.26
SBR/5IO	0.98	0.8	0.78	0.64	−0.34
SBR/10IO	0.88	0.78	0.71	0.67	−0.21
SBR/2PB71	0.97	0.73	0.65	0.6	−0.37
SBR/5PB71	0.98	0.75	0.58	0.55	−0.43
SBR/10PB71	1.01	0.92	0.83	0.8	−0.21

**Table 2:** PCFC data obtained for different elastomer composites containing inorganic pigments.

Compound	pHRR (W/g)	THR_max_ (kJ/g)	HRC (J/gK)
NR	578.7	35.6	578.5
NR/10IO	515.2	32.1	518.6
NR/10PB71	505.4	34.0	498.5
NBR	365.4	33.1	369.9
NBR/10IO	327.5	29.8	330.2
NBR/10PB71	334.4	30.6	331.4
SBR	368.9	42.3	366.5
SBR/10IO	359.2	38.9	356.0
SBR/10PB71	227.2	31.4	238.7

**Table 3:** Formulations of the elastomer composites containing inorganic pigments (in phr).

Compound Name/ Formulation	NR	NBR	SBR	S	ZnO	MBT	Stearin	Iron Ocher	Zirconium Cerulean Blue
NR	100	-	-	2	5	2	1	-	-
NR/2IO	100	-	-	2	5	2	1	2	-
NR/5IO	100	-	-	2	5	2	1	5	-
NR/10IO	100	-	-	2	5	2	1	10	-
NR/2PB71	100	-	-	2	5	2	1	-	2
NR/5PB71	100	-	-	2	5	2	1	-	5
NR/10PB71	100	-	-	2	5	2	1	-	10
NBR	-	100	-	2	5	2	1	-	-
NBR/2IO	-	100	-	2	5	2	1	2	-
NBR/5IO	-	100	-	2	5	2	1	5	-
NBR/10IO	-	100	-	2	5	2	1	10	-
NBR/2PB71	-	100	-	2	5	2	1	-	2
NBR/5PB71	-	100	-	2	5	2	1	-	5
NBR/10PB71	-	100	-	2	5	2	1	-	10
SBR	-	-	100	2	5	2	1	-	-
SBR/2IO	-	-	100	2	5	2	1	2	-
SBR/5IO	-	-	100	2	5	2	1	5	-
SBR/10IO	-	-	100	2	5	2	1	10	-
SBR/2PB71	-	-	100	2	5	2	1	-	2
SBR/5PB71	-	-	100	2	5	2	1	-	5
SBR/10PB71	-	-	100	2	5	2	1	-	10

## Data Availability

The data presented in this study are available upon request from the corresponding author.
